# Vick's Illustrated Guide for 1881

**Published:** 1881-02

**Authors:** 


*Vick's Illustrated Floral Guide for* 1881. Publisher, James Vick, Rochester, N. Y.

This is an elegant book of one hundred and twenty pages; one colored flower plate of petunias, &c, and 600 illustratious, with an engraved likeness of the publisher. It contains descriptions of the best flowers and vegetables, and directions for growing, and is in English or German to suit the different nationalities. Price, 10 cts., which is deducted if seeds are afterwards ordered. Mr. Vick is also the publisher of *the Flower and Vegetable Garden*, 175 pages, 6 with colored plates and 508 engravings. Price, 50 cts. in paper and $1.00 in cloth; also of *the Illustrated Monthly Magazine*, of 32 pages, and one colored plate per number. Price, $1.25 a year. Vick's seeds are considered to be among the most prolific and the best in the country.

